# Tribological Aspects, Optimization and Analysis of Cu-B-CrC Composites Fabricated by Powder Metallurgy

**DOI:** 10.3390/ma14154217

**Published:** 2021-07-28

**Authors:** Üsame Ali Usca, Mahir Uzun, Mustafa Kuntoğlu, Serhat Şap, Khaled Giasin, Danil Yurievich Pimenov

**Affiliations:** 1 Department of Mechanical Engineering, Faculty of Engineering and Architecture, Bingöl University, Bingöl 12000, Turkey; 2 Department of Mechanical Engineering, Faculty of Engineering, İnönü University, Malatya 44280, Turkey; mahir.uzun@inonu.edu.tr; 3 Mechanical Engineering Department, Technology Faculty, Selcuk University, Konya 42130, Turkey; mkuntoglu@selcuk.edu.tr; 4 Department of Electricity and Energy, Vocational School of Technical Sciences, Bingöl University, Bingöl 12000, Turkey; ssap@bingol.edu.tr; 5 School of Mechanical and Design Engineering, University of Portsmouth, Portsmouth PO1 3DJ, UK; khaled.giasin@port.ac.uk; 6 Department of Automated Mechanical Engineering, South Ural State University, Lenin Prosp. 76, 454080 Chelyabinsk, Russia; danil_u@rambler.ru

**Keywords:** Cu-B-CrC composites, tribology, powder metallurgy, optimization, wear rate

## Abstract

Tribological properties of engineering components are a key issue due to their effect on the operational performance factors such as wear, surface characteristics, service life and in situ behavior. Thus, for better component quality, process parameters have major importance, especially for metal matrix composites (MMCs), which are a special class of materials used in a wide range of engineering applications including but not limited to structural, automotive and aeronautics. This paper deals with the tribological behavior of Cu-B-CrC composites (Cu-main matrix, B-CrC-reinforcement by 0, 2.5, 5 and 7.5 wt.%). The tribological characteristics investigated in this study are the coefficient of friction, wear rate and weight loss. For this purpose, four levels of sliding distance (1000, 1500, 2000 and 2500 m) and four levels of applied load (10, 15, 20 and 25 N) were used. In addition, two levels of sliding velocity (1 and 1.5 m/s), two levels of sintering time (1 and 2 h) and two sintering temperatures (1000 and 1050 °C) were used. Taguchi’s L_16_ orthogonal array was used to statistically analyze the aforementioned input parameters and to determine their best levels which give the desired values for the analyzed tribological characteristics. The results were analyzed by statistical analysis, optimization and 3D surface plots. Accordingly, it was determined that the most effective factor for wear rate, weight loss and friction coefficients is the contribution rate. According to signal-to-noise ratios, optimum solutions can be sorted as: the highest levels of parameters except for applied load and reinforcement ratio (2500 m, 10 N, 1.5 m/s, 2 h, 1050 °C and 0 wt.%) for wear rate, certain levels of all parameters (1000 m, 10 N, 1.5 m/s, 2 h, 1050 °C and 2.5 wt.%) for weight loss and 1000 m, 15 N, 1 m/s, 1 h, 1000 °C and 0 wt.% for the coefficient of friction. The comprehensive analysis of findings has practical significance and provides valuable information for a composite material from the production phase to the actual working conditions.

## 1. Introduction

Today, copper (Cu) is widely used for industrial products such as antennas, filaments, contacts and electrodes, due to its properties such as high thermal conductivity, high electrical conductivity and good machinability [1]. Many applications in the manufacturing and electronics industries require material components with improved mechanical properties as well as high thermal and electrical conductivity, high oxidation and corrosion resistance [1,2,3]. Today, scientists are constantly trying to improve the mechanical and workability properties of these materials, which can be produced cost-effectively and with low density [4,5,6,7]. One way to improve the mechanical and machinability properties of copper is by adding a second phase [8]. Composite materials, which are formed by the combination of more than one material, have properties such as high hardness, high strength, low thermal expansion, power damping and excellent wear resistance [9,10]. Metal matrix composites (MMCs) are materials with significantly improved properties [11,12]. It has been stated previously that MMCs—especially copper matrix composites (Cu-MMC)—are the most suitable materials that can be used in the industry for the aforementioned properties [13,14]. The powder metallurgy method (P/M) is a metal forming process which involves mixing elemental or alloy powders to form MMCs [8,15,16]. The P/M method is often preferred due to its advantages such as high production speeds, low cost, the manufacturing of complex shapes, low material loss and high melting temperature [17,18,19,20,21,22]. In addition, ceramic reinforcements have been attracting attention due to their high melting temperature, hardness and corrosion resistance. A number of application fields such as automotive, military and electronics prefer to use ceramics today [23,24]. On the basis of above discourses, CrC was employed as the ceramic reinforcement particle thanks to their superior mechanical properties. In light of the outlined information, this study focuses on the tribology behavior of Cu-B-CrC composites.

Despite Cu carrying significant properties for several types of engineering materials utilized in the automotive, electric and electronic sectors, there are limitations for its applications. To overcome these restrictions, the key factor is to select the accurate additives to improve the its mechanical properties [25]. An important improvement has been procured by CrC particles in terms of material hardness, abrasion resistance and tensile strength by the effect of superior hybrid properties of this ceramic such as wear resistance, thermal stability and corrosion resistance [26]. On the other hand, the addition of boride to the material structure of different types of metal matrix [27,28,29,30] in the past improved their mechanical properties. Many initiatives have been presented in the past for better copper matrix properties. One of the main reinforcement materials is graphene due to its excellent heat and electrical conductive properties [31,32,33]. Graphene is a single layer of carbon atoms which are stacked together in a honeycomb-like lattice structure. For example, Cao et al. [34] experimentally tested graphene and tungsten additives under different loads according to wear and tribological aspects. Important improvements were obtained in this way, inspired by researchers, after Ma and Lu [35] addressed the influence of sliding distance on the tribological behavior of Cu-based composites reinforced by graphene. A work conducted by Mai et al. [36] selected nickel as the additive element in different ratios. According to the results of this study, a critical level of graphene brings significant improvements to the copper matrix. Xiao et al. [37] evaluated the tribological behavior of the Cu matrix reinforced by MoS_2_ and AISI 52100 steel in order to find the optimum reinforcement ratio of MoS_2_. It was verified that MoS_2_ is an effective lubricant for copper matrix composites against steel. This situation was attributed to the friction coefficient decreased by adding 20 vol% of the MoS_2_. Wu et al. [38] studied the effect of Ti_2_SnC reinforcement on Cu considering its electrical and mechanical properties. According to the authors, the coefficient of friction and wear rate of the Cu matrix was improved significantly. Rajkumar and Aravindan [39] measured the size effect of the graphene particles on Cu-based composites, and found that nano-sized particles provide an important improvement in the wear and friction properties. Su et al. [40] investigated the surface integrity and tribological aspects of copper-based composites by adding graphene particles. Graphene reinforcement procured considerable impact on these properties from the improvement perspective. Xiao et al. [41] researched friction and wear properties for Cu composites. The tribological performances demonstrated little sensitivity to friction cycles in the determined temperatures. Tang et al. [42] compared the tribological properties of pure copper and carbon-fiber-strengthened copper matrix composites and found that the latter showed superior properties. Kumar et al. [43] evaluated the performance of Cu-based, copper–tin and MoS_2_-reinforced composites according to their microstructures and tribological conditions. Accordingly, the developed composite demonstrated a lower coefficient of friction and wear rates compared to the prepared composites. Zhao et al. [44] investigated the coefficient of friction, wear rate and microstructures of tungsten added composites. Superior tribological properties were obtained by the formation of these tribo-films. Huang et al. [45] indicated that Cu-based composites can be improved by adding carbon nanotubes by means of mechanical and tribological aspects. In the work of Zou et al. [46], Cu and graphene were prepared as the mother matrix reinforced by SiO_2_ particles in order to improve the tribological and mechanical properties. According to the authors, the addition of SiO_2_ leads to increasing friction stability and friction coefficient, and decreasing wear rate. Rajkumar et al. [47] proposed hybrid composites fabricated by Cu and reinforced by TiC and graphene particles. Better tribological properties were obtained with these ceramic particles. Zhan and Zhang [48] also produced hybrid composites by adding SiC and graphene particulates. They obtained higher wear and tribological properties by the ceramic reinforcements. Rajkovic et al. [49] used Al_2_O_3_ ceramics in order to develop the main Cu matrix which was the same with Hwang et al. [50]. They reported the superior properties and positive effect of the ceramic additives. Triantou et al. [51] evaluated different ratios of Al_2_O_3_ ceramics and obtained higher tribological characteristics. Sap [52] used cobalt and titanium powders in order to strengthen the Cu-based matrix by means of microstructural and mechanical aspects. Accordingly, the reinforcement ratios were relatively more effective compared to the other parameters. Uzun and Cetin [26] added cobalt and chrome carbide to the Cu-based matrix for the evaluation of microstructure characterization. The reinforcement particle ratio of wt. 10% was the optimum reinforcement ratio for this study. Gong et al. [53] added SiO_2_ and CrC to the Cu-based composites for improved microstructural and tribological properties. When looking into the open literature, it can be seen that there is a handful of studies which looked into the influence of adding different types of additives on the tribological performance of Cu-based composites. Uzun et al. [54,55] researched the influence of different ratios of CrC particulate reinforcements on the microstructure, hardness and wear properties of a Cu-based matrix. Gautam et al. [56] addressed tribological behavior of Cu–Cr–SiCp in situ composites considering wear rate, volume loss, surface roughness and coefficient of friction. Briefly, Cu provides convenient conditions for particle addition to a wide range of materials. Increasing demand on the advanced mechanical and tribological performance of the end product has led researchers and manufacturers to discover different new-generation composite materials with specific high strength, lightweight properties and enhanced surface aspects. In this context, various studies have been performed on the mechanical and tribological properties of different metal-based matrix composites produced by powder metallurgy routes. However, no published study has been encountered that deals with the effect of production parameters on tribological properties, both experimentally and statistically, particularly herein the Cu-B-CrC system.

Despite the fact that there has been a significant amount of published literature about the tribological aspects of Cu-based composites, this study is the first considering the influence of adding B-CrC as a reinforcement element. The paper focuses on the tribological behavior of Cu-B-CrC composites produced by powder metallurgy. Taguchi-based orthogonal array (L_16_) was adopted to the experimental design using four levels of each of the following: sliding distance, applied load and reinforcement ratio. Moreover, two levels of the following: sliding velocity, sintering time and sintering temperatures were used The aim is to evaluate the tribological behavior, coefficient of friction and wear rate of Cu-B-CrC composite and using Taguchi’s signal-to-noise ratios for optimization, with ANOVA for statistical analysis and 3D plots for changing parameter effects.

## 2. Materials and Methods

### 2.1. Composite Materials Production Process

**Figure 1:**
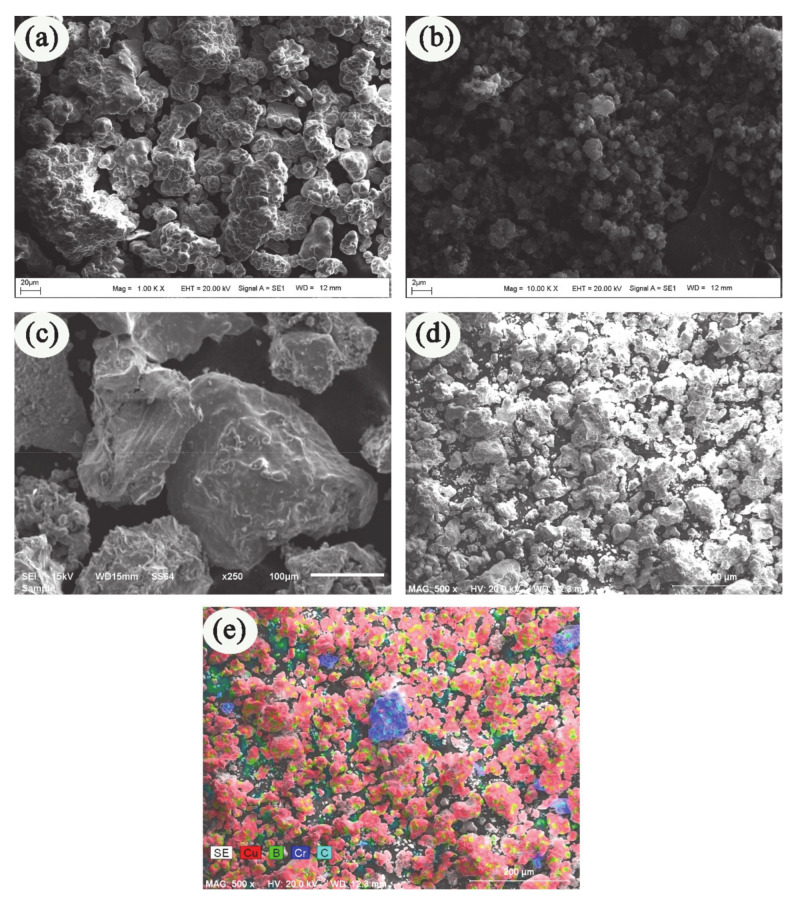
SEM images of powders used in composite material production (**a**) Cu [26] (**b**) B (**c**) CrC (**d**) mixed powder (5 wt.%), and (**e**) EDX mapping analysis of Figure 1d.

High commercial purity Cu powders were used as the main matrix for the composite materials produced in this study. Elemental powders of Cu (<45 μm, irregular particle shapes), and B (~2 μm, irregular particle shapes) and ceramic powder of Cr_3_C_2_ (~300 μm, prismatic morphology) were purchased from Nanografi Nanotechnology, Co. Ltd., Ankara, Turkey. The purity rates of the powders used are greater than 99%. Boron powders, known for their resistance to heat at high temperatures, and Cr_3_C_2_ powders, which provide excellent abrasion resistance, were used as reinforcement materials. The reinforcing particles used can improve certain properties (such as wear resistance and hardness) of the base matrix used for the composite material. Scanning Electron Microscope (SEM) (JEOL Ltd., Tokyo, Japan) images of the main matrix and reinforcement particles used in this study are given in Figure 1.

The composite materials used in the experiment were produced by the powder metallurgy method. Commercial grade B-Cr_3_C_2_ supplement powder particles were added to the Cu main matrix at a ratio of 0–2.5–5–7.5 wt.%, as shown in Table 1. Elemental powders were mixed for 4 h at a rotational speed of 50 rpm with the help of a turbula to distribute homogeneously. The powders that became homogeneous were pressed by the cold pressing method under 600 MPa pressure in a hydraulic press (Hidrometal, Konya, Turkey).

**Figure 2:**
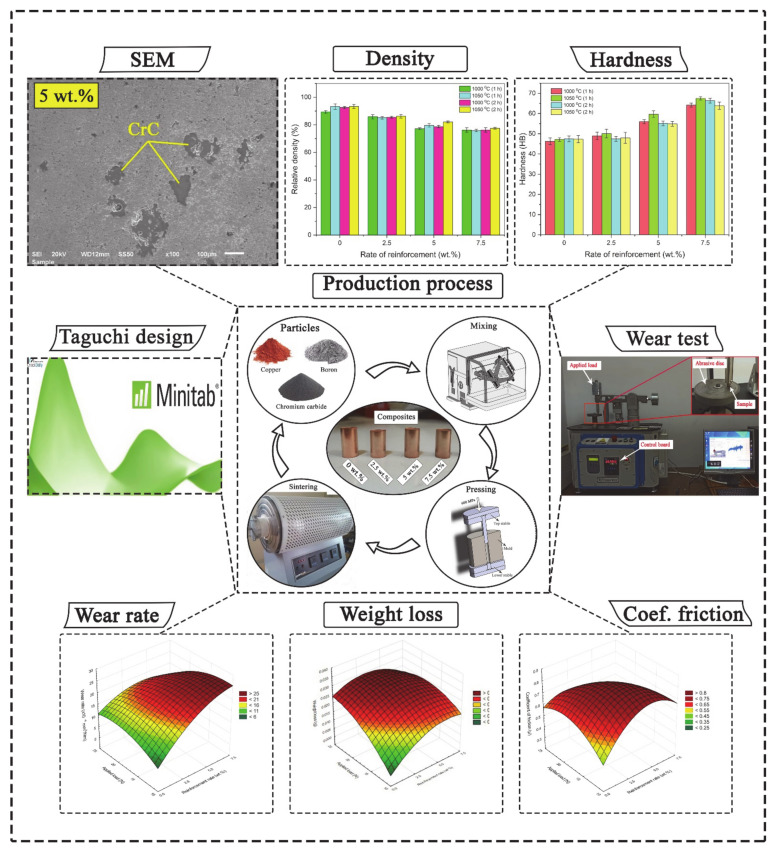
The experimental scheme the experimental scheme.

Pressed samples were sintered under protective argon atmosphere (dry argon having a dew point of −55 °C) in a Protherm HLF-50 sinter furnace (Prothermfurnaces, Ankara, Turkey) according to L_16_ Taguchi orthogonal array. In the sintering process, 1000 °C and 1050 °C, 1 h and 2 h parameters were selected for the temperature. After sintering, 12 mm diameter and 30 mm length cylindrical test specimens were obtained. The experimental scheme is shown in Figure 2.

### 2.2. Microstructural Evaluation

In order to obtain microstructural images of the produced samples, the P200-400-600-800-1000-1200 grid was sanded with paper backing SiC discs, respectively. After sanding, 3 µm diamond suspension was used for polishing. The etched samples, which were etched by %5 Nital and ammonium persulfate (10 g (NH_4_)S_2_O_3_ + 90 g deionized water) and their dilution with ethanol, were made ready for SEM. The samples were cleaned with ethyl alcohol to avoid any residue on the surface of the samples. Metallographic analysis of the samples was carried out on the JEOL JSM 6510 branded SEM device (JEOL Ltd., Tokyo, Japan).

### 2.3. Density and Hardness Measurements

**Figure 3:**
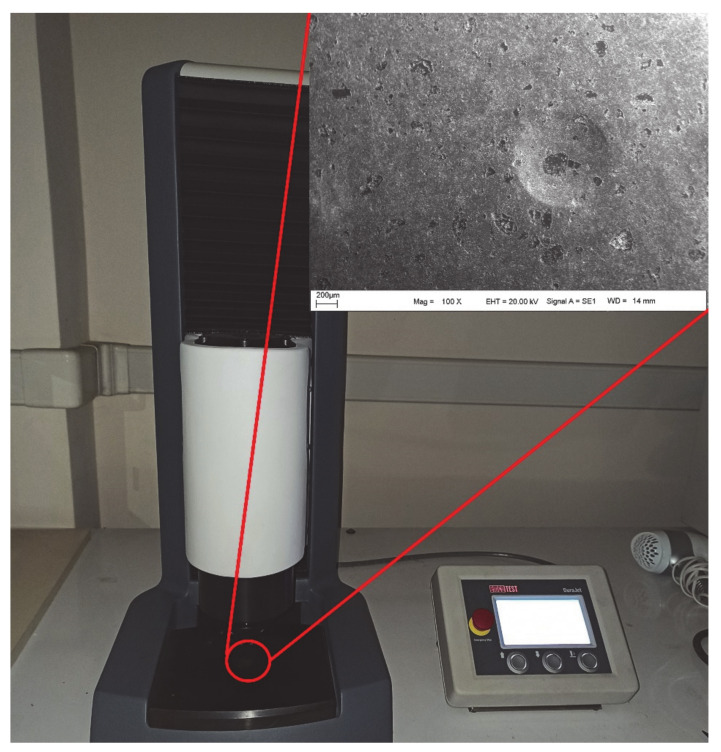
Brinell hardness test.

After the microstructural characterization of the powders, the mechanical properties of the sintered samples were determined using density and Brinell hardness measurements. According to the information available in the literature survey [57,58], the Brinell hardness method was selected because it has high accuracy and repeatability for metals and their alloys. Plus, the indenter spherical ball used by different sizes allows more effortless measurement on the material surface than Vickers hardness. These properties of the Brinell hardness technique makes it highly influential in evaluating the hardness relationships between the matrix and reinforcement element in a multiphase material system. In order to obtain information about the pores formed in the produced composites, the relative density of the samples was determined. The Brinell hardness method was used to evaluate the macro hardness of the produced composite materials. The hardness measurements were carried out using a BMS hardness tester (Bulut Makina, Kocaeli, Turkey) by applying a 10 kg load to the flat and polished surface of the samples for 10 s (Figure 3). For each sample, the hardness measurement was repeated five times and the average of the five readings was reported in the results.

### 2.4. Taguchi-Based Experimental Design and Optimization with Signal-to-Noise Ratios

Taguchi is a form of experimental design methodology which is often used to reduce the number of tests required when the experiment contains a large number of factors and levels [59]. Taguchi is a frequently utilized method in many industrial applications and engineering fields. It also supplies reliable and high accuracy results. Further, it is a promising approach for tribological aspects which has been applied by the authors recently [60,61]. In this context, a Taguchi-based statistical design was chosen in the present study to reduce the number of runs (tests) which are formed from weight loss, wear rate and the coefficient of friction. In addition, the optimization using Taguchi is achieved using signal-to-noise ratio–smaller-the-better principle, as shown in Equation (1) [62].

(1) $${\mathrm{Signal}-\mathrm{to}-\mathrm{noise}\;}_{\mathrm{smaller}\;\mathrm{is}\;\mathrm{the}\;\mathrm{better}}=-10\log\left[\frac{1}{n}\;\sum_{i=1}^{n}y_{i}^{2}\;\right]$$

where *y* shows the responses of the machinability characteristics, for a trial condition repeated several times.

### 2.5. Wear Analysis

Wear tests of Cu-B-CrC composite samples produced at different ratios were performed on the TURKYUS model pin-on-disc tribometer device and optimized by the Taguchi method. The Taguchi method was used to reduce the number of runs required in the wear test. The diameters of the samples produced for the wear test were reduced to 10 mm. The disc used as an abrasive was produced from the AISI D2 tool steel by the wire erosion method and its surfaces were hardened by nitriding. An abrasive disc wear test was applied to test specimens and these samples with a diameter of 10 mm and a length of 30 mm were prepared according to the ASTMG99-95a standard. The wear test was started with a circular motion by contacting the test specimens on the rotating disc surface. Before the test, the surfaces of the disk and cylindrical test specimens were cleaned by washing them with ethyl alcohol. The friction force produced during the wear test was measured using a strain gauge sensor. The fixed variables used for testing were as follows: the disc diameter at which the wear test was carried out was 96 mm, the disc speed was 200 and 300 RPM, the sliding distance was 1000–1500–2000–2500 m, the sliding speed was 1–1.5 m/s and the applied loads were 10, 15, 20 and 25 N. Weight losses and variation of the friction coefficient were investigated. All wear tests were performed under dry conditions in a normal laboratory atmosphere (55–70% relative humidity, 20–25 °C).

## 3. Results and Discussion

### 3.1. SEM-EDX Analysis

**Figure 4:**
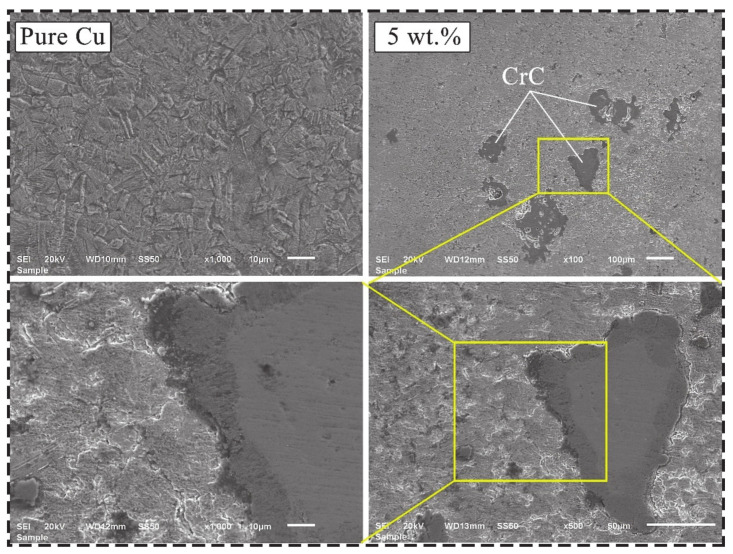
SEM micrographs of pure Cu [63] and composites produced at 5 wt.%.

SEM micrographs showing the microstructures of composites produced at different ratios are shown in Figure 4. When the microstructures of the composites are examined, it is seen that there are light and dark regions. The light-colored regions represent the copper main matrix and the dark-colored regions represent the reinforcement particles. Sap [8] produced composite materials by adding different proportions of Co-Mo hybrid powder particles into the Cu main matrix. She reported that light and dark areas were detected in the composite samples produced. The sharp edges of the additive particles directly affect the formation of porosity. Thus, it can cause a decrease in the relative density. However, it was thought that the main matrix could improve the mechanical properties due to better adhesion to the edges. CrC particles were detected in the copper-rich microstructure. Boron was not seen in the microstructure due to its smaller grain size. In general, it can be said that there is a homogeneous distribution in the microstructure. Additionally, no agglomeration was found.

**Figure 5:**
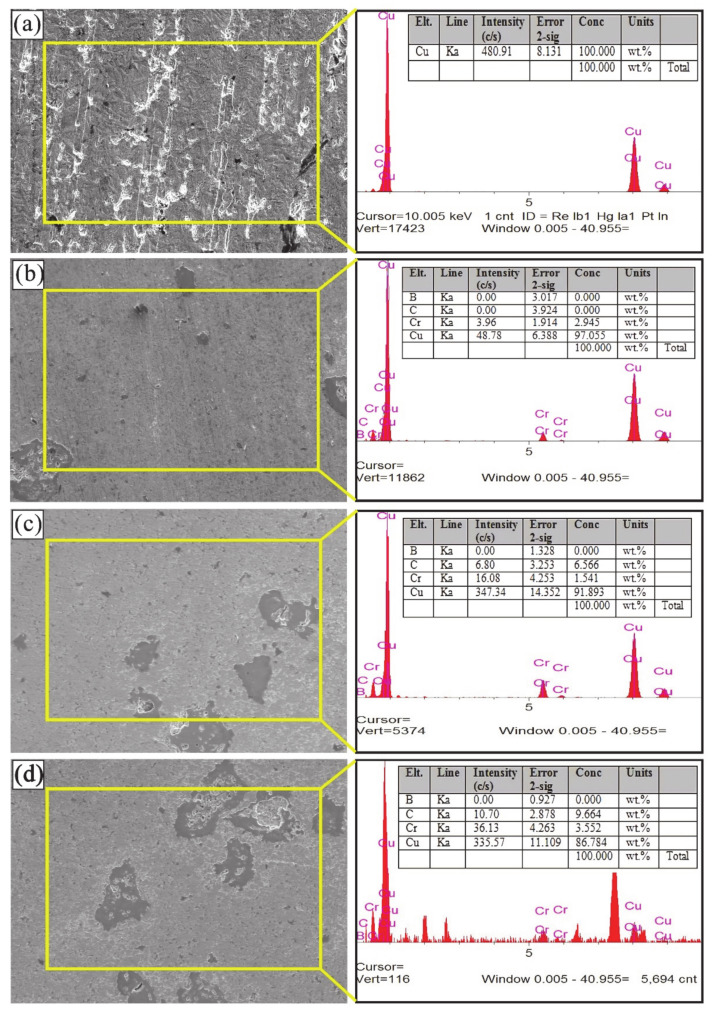
SEM-EDX micrographs of composites produced at different ratios: (**a**) pure sample, (**b**) 2.5 wt.% B-CrC, (**c**) 5 wt.% B-CrC, (**d**) 7.5 wt.% B-CrC.

SEM/EDX analysis of composite samples with different reinforcement ratios is shown in Figure 5. In Figure 5a, it is seen that the copper peaks are high. Since the sintering process was carried out with protective argon gas, no oxygen or oxidation was found on the sample surfaces as a result of the analysis. In addition, no undesirable compounds were encountered. In the EDX analysis of composites, the ratios of the elements in the grain boundaries and the main matrix support each other.

### 3.2. Density and Hardness Analysis

**Figure 6:**
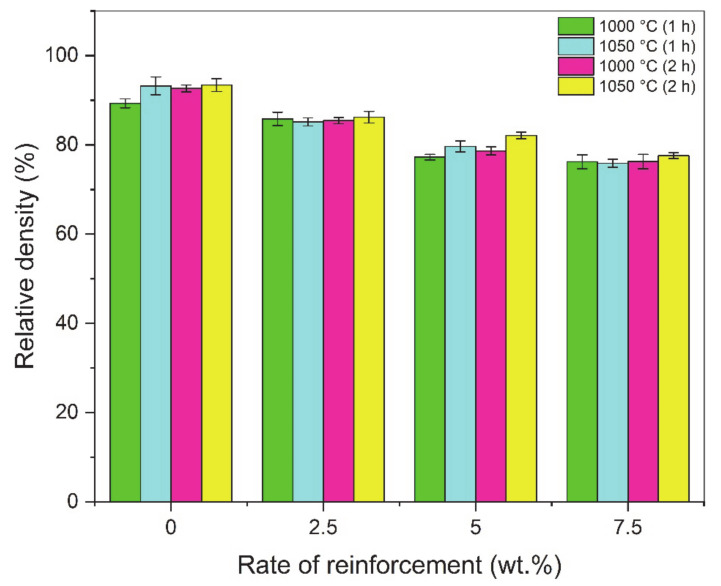
Relative density graph of composites sintered at different times and temperatures.

The relative density graphs of composites sintered at different times and temperatures are shown in Figure 6. When the graph is examined, it is seen that the highest density values are in the pure copper sample. In general, it was determined that the relative density values increased with increasing sintering time and temperature [14]. The amount of pores formed during sintering directly affects the mechanical properties of the material [64]. Especially during sintering, neck formation and diffusion rate are directly related to sintering temperature. Therefore, the highest density value (93.4%) was found in the sample sintered at 1050 °C for 2 h. The increase in sintering time and temperature increases the relative density.

**Figure 7:**
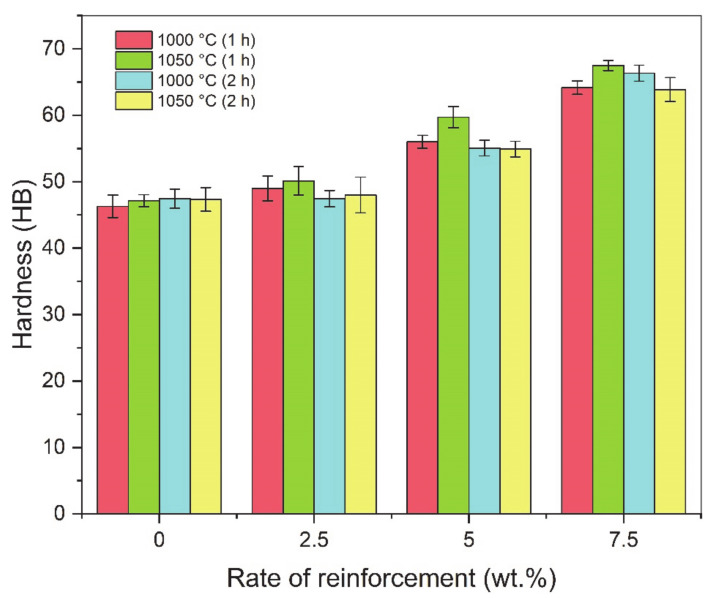
Hardness graph of composites sintered at different times and temperatures.

The hardness of the composites produced using different sintering times and temperatures is shown in Figure 7. The results showed that the hardness values were higher in the 7.5 wt.%-reinforced samples. It was determined that the hardness increased with the increase in the weight of the hardness reinforcement particles, which are the indicators of resistance to plastic deformation. In general, it was observed that the samples sintered at 1050 °C for 1 h had higher hardness values. The highest hardness value (67.48 HB) among all samples was found in the 7.5 wt.%-reinforced sample, which was sintered for 1 h at 1050 °C. Therefore, it can be said that the sintering temperature have a greater effect on hardness than the sintering time.

### 3.3. Optimization and Graphical Analysis for Wear Rate

As mentioned previously, four levels of sliding distance (1000–1500–2000–2500 m), applied load (10–15–20–25 N) and reinforcement ratio (0–2.5–5–7.5 wt.%), two levels of sliding velocity (1–1.5 m/s), sintering time (1–2 h) and temperature (1000–1050 °C) were adopted to the Taguchi experimental design. Table 2 outlines these inputs and the obtained results, namely weight loss (g), wear rate and coefficient of friction (µ). A total of 16 experiments were performed, which are further analyzed for each output, respectively.

**Figure 8:**
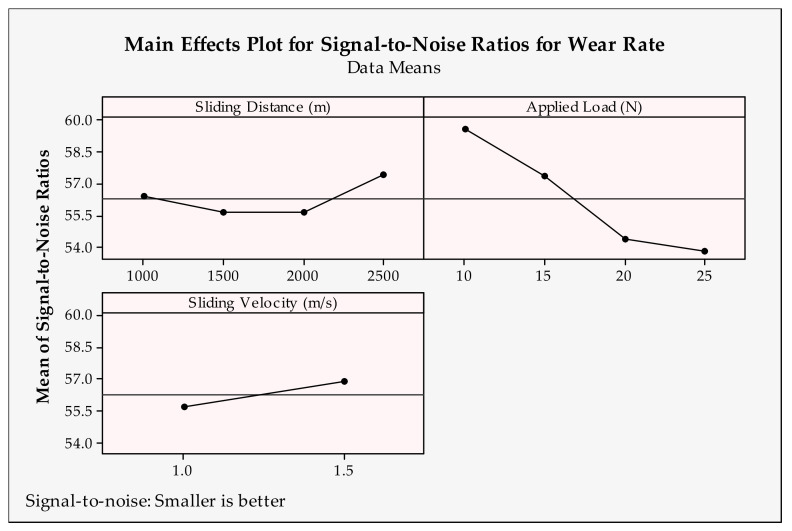
Signal-to-noise ratios of wear rate for tribological parameters.

**Figure 9:**
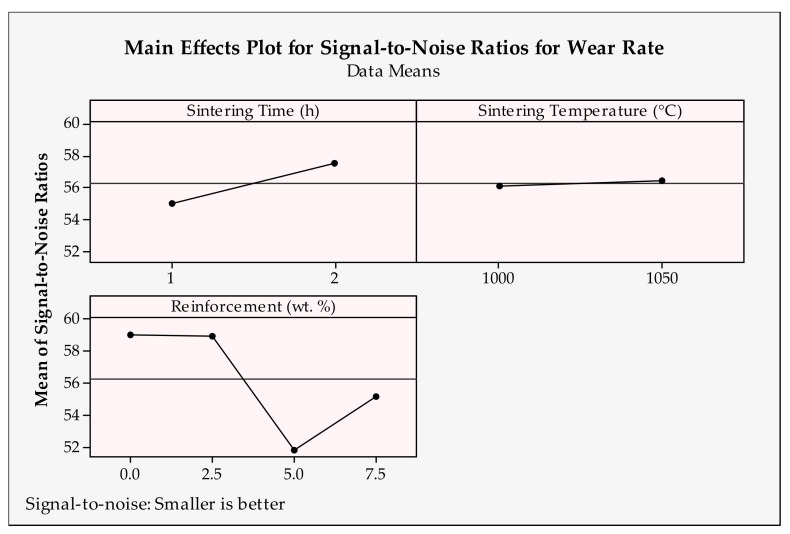
Signal-to-noise ratios of wear rate for production parameters.

Wear rate shows the amount of wear of the materials used in the experiments. It is important to measure the wear characteristics of the engineering parts. Therefore, the optimum conditions for procuring the minimum wear rate value are required. The main effects plots calculated by signal-to-noise ratios for wear rate results are depicted in Figure 8 and Figure 9, which show the optimum solutions for each input parameter. Accordingly, the highest levels of parameters (2500 m, 1.5 m/s, 2 h and 1050 °C) and lowest values of other parameters (10 N and 0 wt.%) should be selected for the minimum wear rate. 

When looking at the tendency of the parameter levels, the specific wear rate increases with the increase in sliding distance, reinforcement ratio and applied load first, then decreases to the maximum value of them. A higher wear rate is expected due to harsh tribological conditions with the increase in the sliding distance and applied load. This result was found for the composite materials before [65]. A larger load and long-distance produce a high amount of heat and pressure at the contact zones, which make easier wear conditions. The sliding distance produces the best wear conditions at the maximum value. This behavior can be evaluated as the lubricating effect of carbon atoms in the wear zone of the worn reinforcement material CrC. Additionally, a higher reinforcement ratio increases the hardness of the material and aggravates the wearability [66]. There is an unexpected disposition from 5 wt.% to 7.5 wt.%, which defines better wear characteristics for the high amount of reinforcement. This can be attributed to the wear tests. It can be said that the graphene additives in the materials form a significant amount of lubricating film in the later stages of the wear test [67]. In addition, higher velocity prevents the favorable contact conditions between the pin and disk materials and causes a decrease in wear [68]. As a representative parameter group, the sintering time and temperature influence interfacial bonding and resultant structural integrity [69]. Therefore, it is critical to determine the optimum temperature and time during the sintering operation in order to reach better material structures. Here, it can be said that using the samples with a 2.5 wt.% reinforcement ratio, higher sintering parameters can be operated for Cu-B-CrC composites.

**Figure 10:**
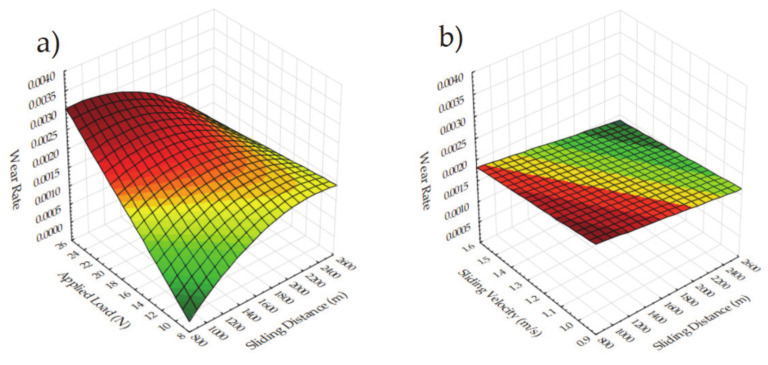

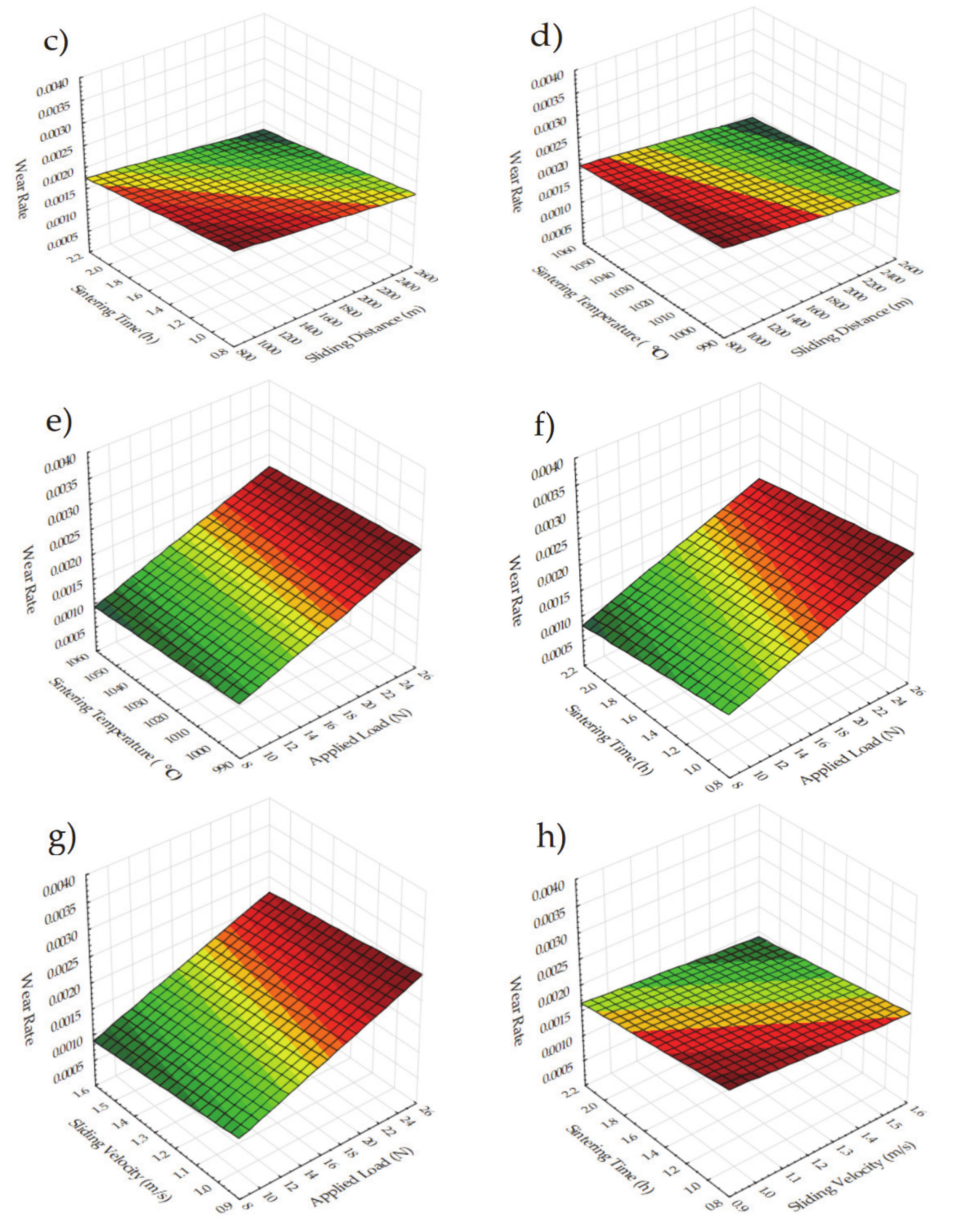

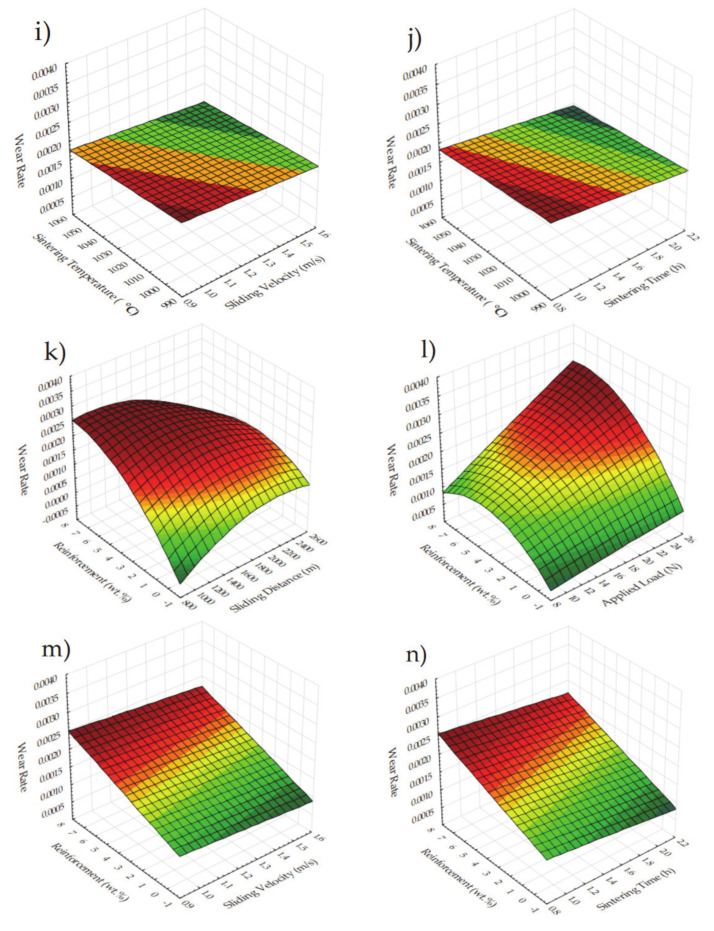

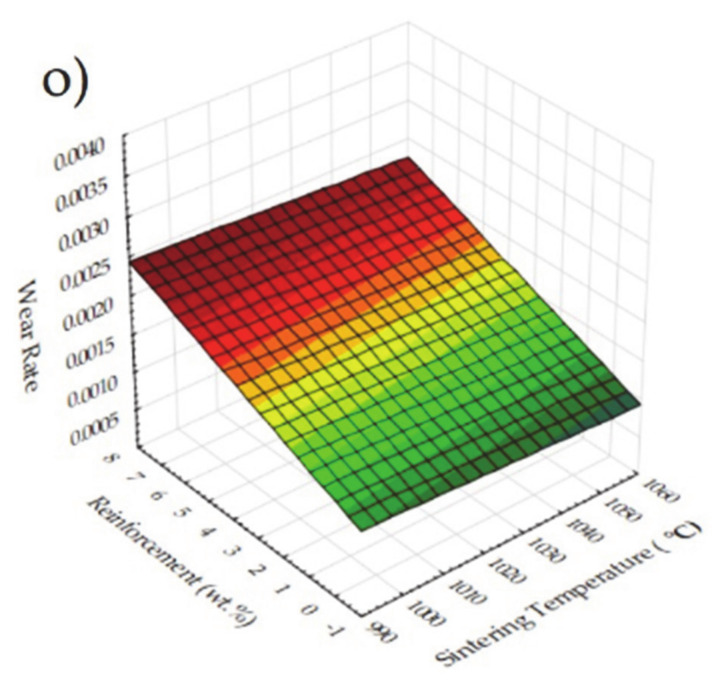
3D surface plots for wear rate for the effect of (**a**) Sliding distance and applied load (**b**) Sliding velocity and sliding distance (**c**) Sintering time and sliding distance (**d**) Sintering temperature and sliding distance (**e**) Sintering temperature and applied load (**f**) Sintering time and applied load (**g**) Sliding velocity and applied load (**h**) Sintering time and sliding velocity (**i**) Sintering temperature and sliding velocity (**j**) Sintering temperature and sintering time (**k**) Reinforcement and sliding distance (**l**) Reinforcement and applied load (**m**) Reinforcement and sliding velocity (**n**) Reinforcement and sintering time (**o**) Reinforcement and sintering temperature.

Figure 10 shows the 3D plots for a wide range of design parameters and their combined impact on the wear rate. When increasing the applied load, the wear rate shows an increasing trend irrespective of the second parameter, which is depicted in Figure 10a,e–g,l, due to the elevated contact between the pin and disk. Additionally, sintering parameters have a minor impact on the wear rate, as can be seen in Figure 10e,f,m–o. It should be noted that some parameter couples have a minor effect which is accepted as unimportant, represented in Figure 10, i.e., Figure 10b–d,h–j. The combinations of sliding distance and velocity, and sintering time and temperature have less effect on the wear rate. A slight wear rate reduction can be observed when looking at the higher sintering time and sliding velocity at Figure 10f,g, respectively. Increasing wear rate can be seen in samples with high reinforcement, as shown in Figure 10k–o, except for the highest ratio. As explained before, it is thought that this situation occurs owing to the lubricating effect of CrC in the material structure. Cu is a relatively porous material and is frequently preferred as a plain bearing. Due to the fact that the worn structures partially fill the pores less, it can be evaluated that the C in the eroded CrC acts as a better lubricant on the surface [26]. Similar mechanisms for all parameters were mentioned before, so it is unnecessary to address them again. In summary, the sliding distance, applied load and reinforcement ratio contribute most to the wear rate during tribological tests of Cu-B-CrC composites.

### 3.4. Optimization and Graphical Analysis for Weight Loss

**Figure 11:**
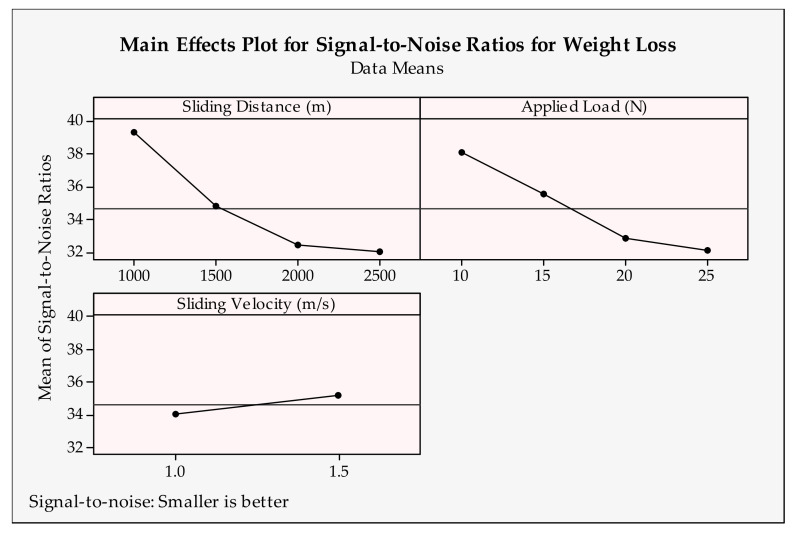
Signal-to-noise ratios of weight loss for tribological parameters.

**Figure 12:**
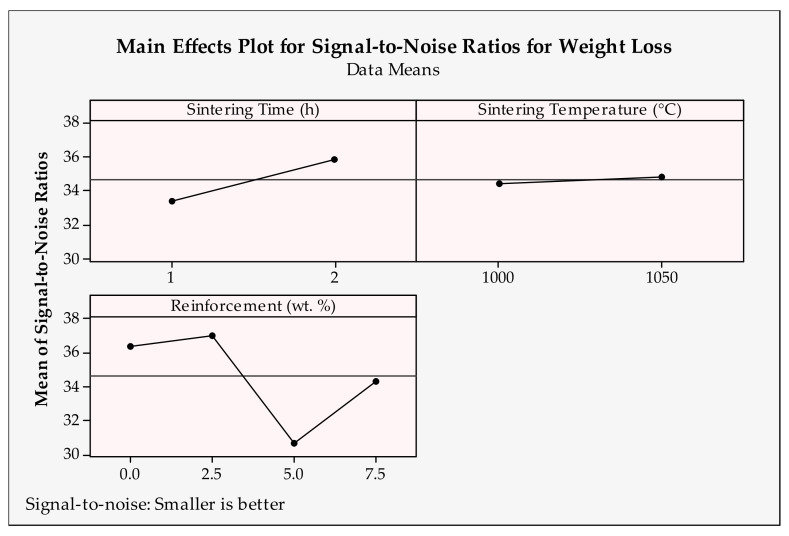
Signal-to-noise ratios of weight loss for production parameters.

The main effects plot of design parameters for weight loss is depicted in Figure 11 and Figure 12. Here, similar to the wear rate results, a smaller is better type objective function was applied. Additionally, according to this, higher signal-to-noise ratios, namely, first levels of sliding distance, applied load (1000 m, 10 N) and second levels of sliding velocity, sintering time, sintering temperature and reinforcement ratios (1.5 m/s, 2 h, 1050 °C and 2.5 wt.%) need to be chosen for the best weight loss result. According to the figures demonstrated, decreasing the sliding distance and applied load produces less weight loss. As expected, a higher load and longer distance cause bigger losses. This phenomena was observed in a study about tribological behavior composites [70]. As observed in the wear rate results, the second level of sliding velocity causes less weight loss [68]. Sintering time and temperature are the functions of plastic deformation [71]. Thus, they mostly affect the matrix reinforcement interface and bonding between particles [72]. Insufficient sintering time and temperature cause a lack of diffusion activity and result in necking and at the later stages turn into particle consolidation. In addition to that, sintering time and temperature may cause coarsened grain; thus, the strength of the material may be negatively affected. Therefore, it can be said that second levels of sintering parameters seem to give minimal weight loss. According to the reinforcement ratios, the best value is 2.5 wt.%. This can be attributed to the better structural integrity of the samples fabricated at this ingredient. Despite the fact that the hardness of the samples increases with a higher reinforcement ratio, it is thought that the additives create a lubricating effect between the surfaces. Seemingly, the findings for weight loss are compatible with the wear rate results.

**Figure 13:**
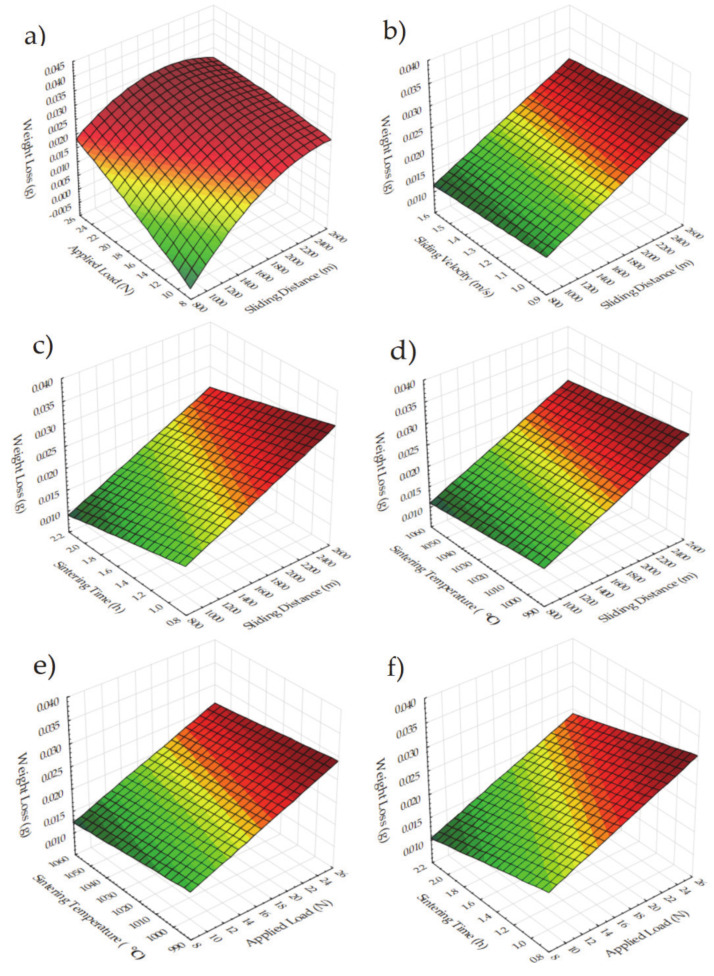

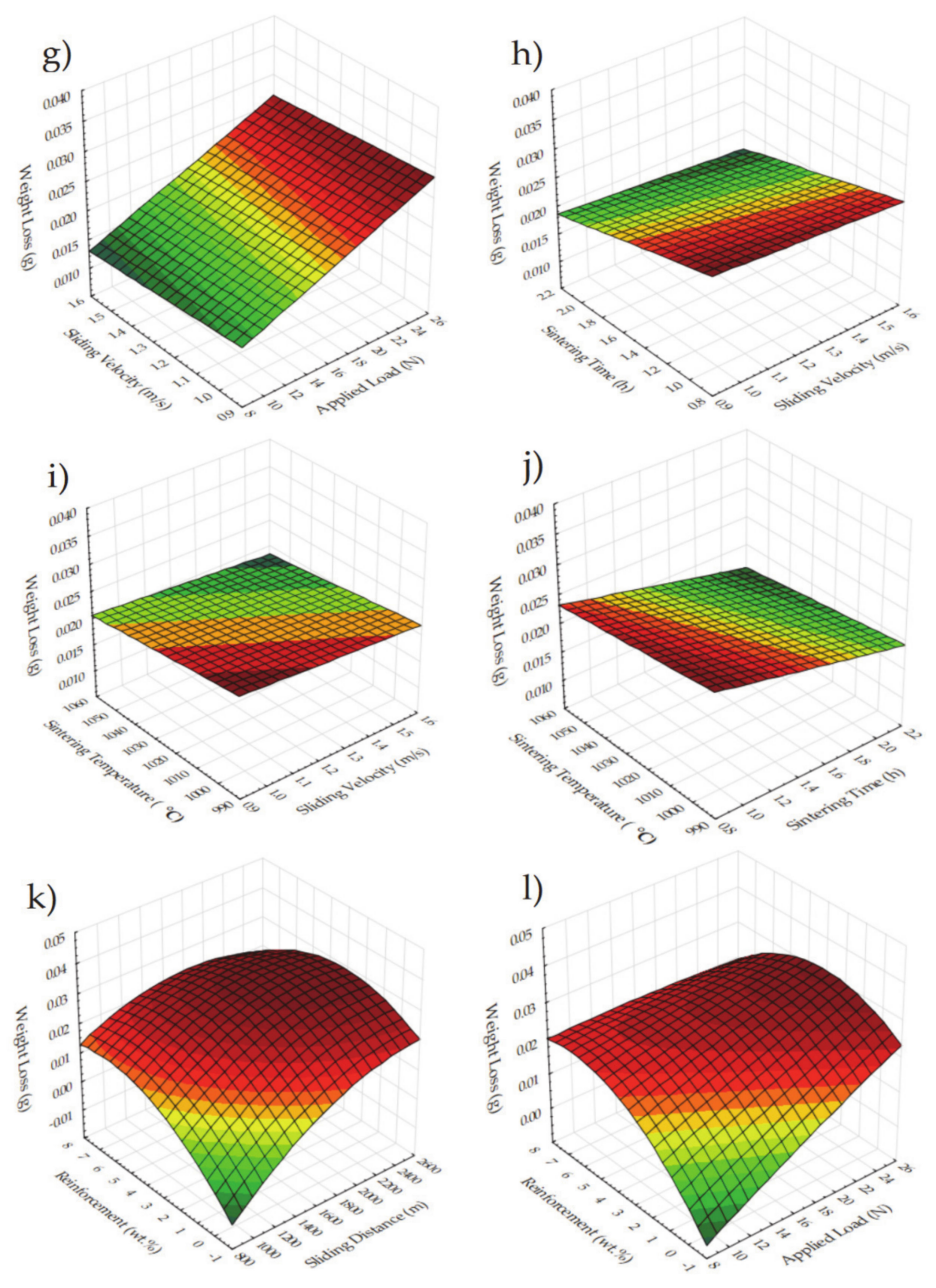

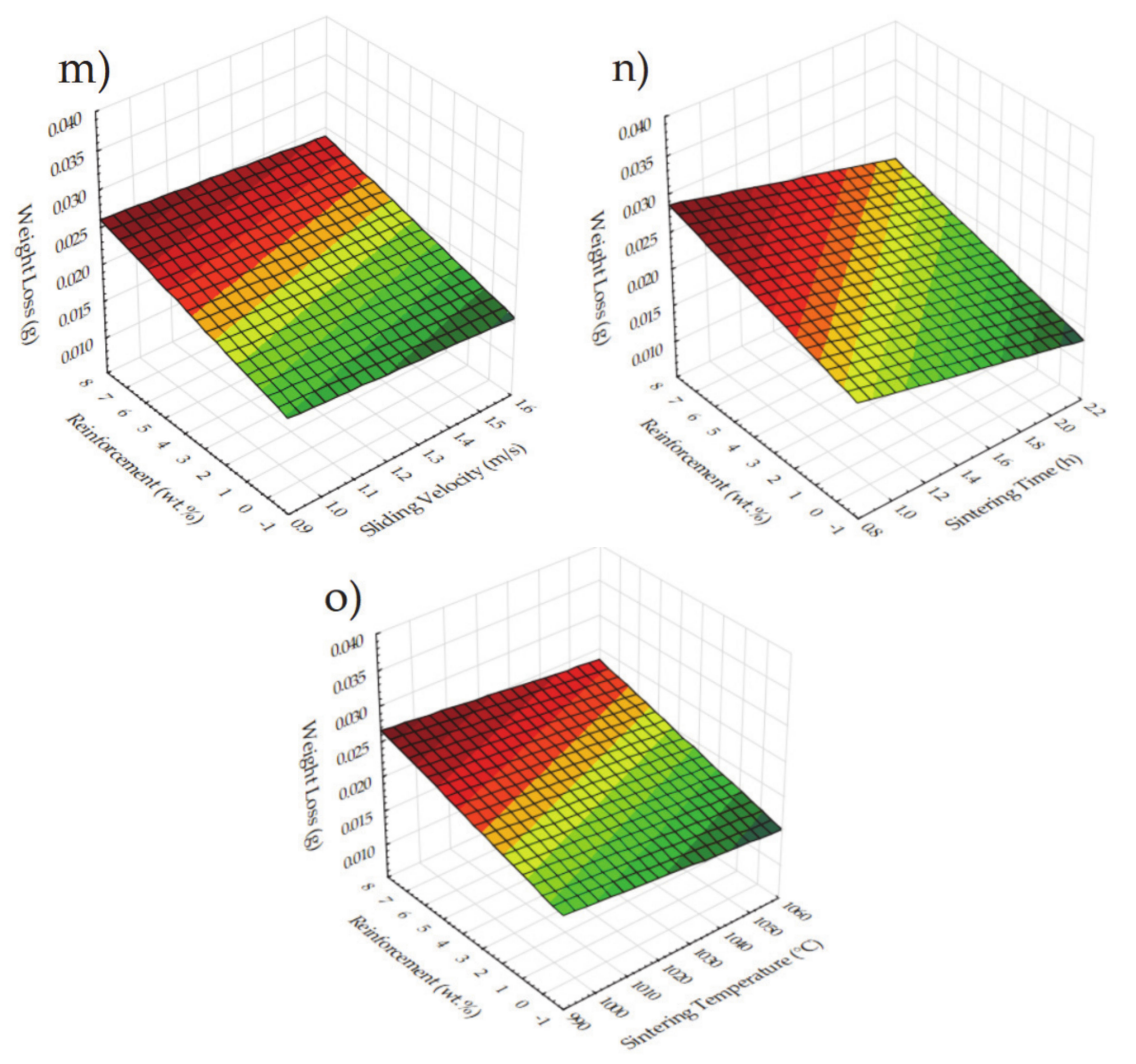
3D surface plots for weight loss for the effect of (**a**) Sliding distance and applied load (**b**) Sliding velocity and sliding distance (**c**) Sintering time and sliding distance (**d**) Sintering temperature and sliding distance (**e**) Sintering temperature and applied load (**f**) Sintering time and applied load (**g**) Sliding velocity and applied load (**h**) Sintering time and sliding velocity (**i**) Sintering temperature and sliding velocity (**j**) Sintering temperature and sintering time (**k**) Reinforcement and sliding distance (**l**) Reinforcement and applied load (**m**) Reinforcement and sliding velocity (**n**) Reinforcement and sintering time (**o**) Reinforcement and sintering temperature.

Weight loss becomes an important issue, especially for the components that work as tandem parts under harsh tribological conditions. As outlined in explanations according to Figure 12, 3D plots for the weight loss are presented here in Figure 13. Due to the weight loss being affected by the wear conditions, similar trends according to different parameter combinations are expected. Figures belonging to the applied load and sliding distance (Figure 13b–g,l) show that increasing values also increase the weight loss. Then, the increase in the weight loss up to a certain value and the decrease with reinforcement can be seen, especially in Figure 13k,l. It is useful to see the linear and quadratic models of reinforcement here for a better description of its effect on weight loss. 

### 3.5. Optimization and Graphical Analysis for Coefficient of Friction

**Figure 14:**
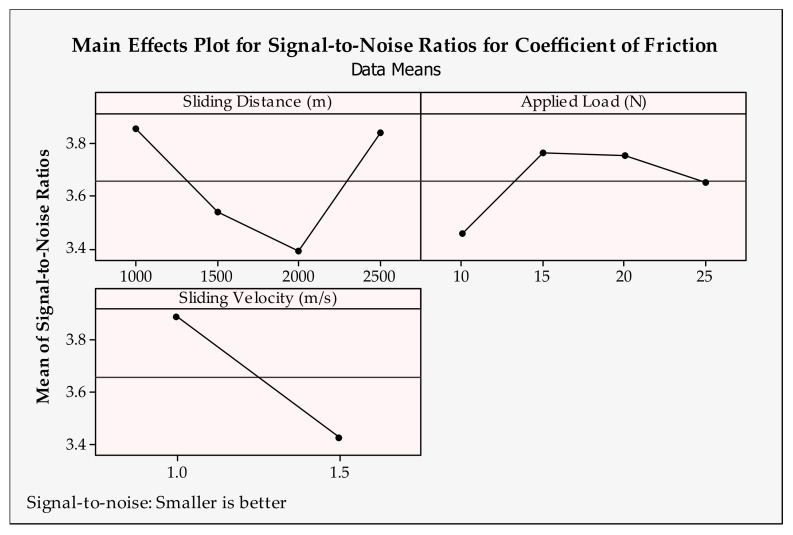
Signal-to-noise ratios of coefficient of friction for tribological parameters.

**Figure 15:**
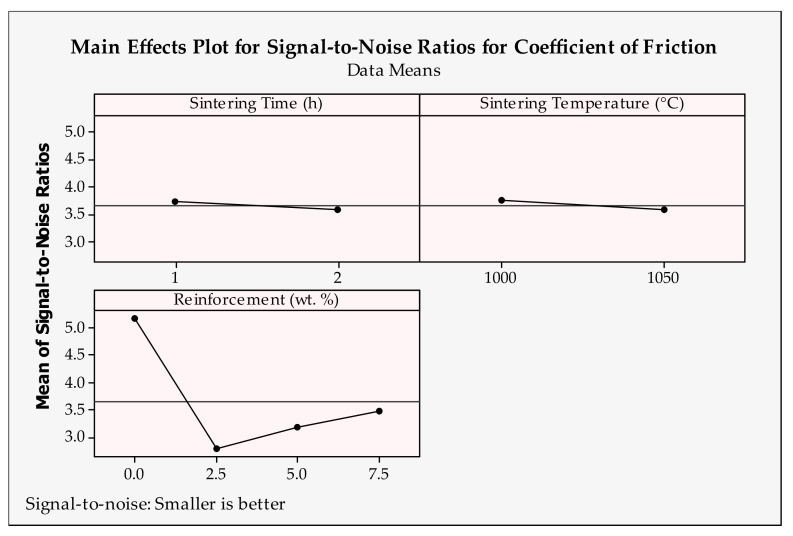
Signal-to-noise ratios of coefficient of friction for production parameters.

The coefficient of friction can be defined as the compelling force between the contacted surfaces. In the tribological aspect, high-strength materials are hard to be worn and therefore tend to produce much more friction when in contact with other materials. According to the main effects plot in Figure 14 and Figure 15, the smallest coefficient of friction can be obtained by applying the following levels for each of the input parameters: sliding distance = 1000 m, applied load = 15 N, sliding velocity = 1 m/s, sintering time = 1 h, sintering temperature = 1000 °C and reinforcement ratio = 0 wt.%.

With increasing sliding distance, the material is subjected to greater abrasive impact and therefore, an elevated coefficient of friction is expected. This effect can be seen from 1000–2000 m in Figure 6. However, up to the 2500 m sliding distance, better frictional conditions are observed, and the coefficient of friction is reduced. This situation is attributed to the self-lubrication property of the reinforcement particles. The sliding distance may have a fluctuating effect on the coefficient of friction under different applied loads and composites, seemingly [65]. When looking at the applied load, from 15 N to 25 N, a gradual increase can be seen in the coefficient of friction. However, at 10 N loads, the highest frictional force appears. A similar observation was reported by Zhang et al. [73]. Seemingly, the applied load has little effect on the coefficient of friction compared to other tribological parameters. In addition, higher velocity also expedites the coefficient of friction and heat generation at the surfaces and wear rate. Therefore, it is understandable that the lower level of sliding velocity is found to be more efficient. This is in line with the results of [73,74]. As mentioned before, the sintering time and temperature have a great effect on the material structure and density [69]. Here, sintering properties have less effect on the coefficient of friction and lower levels are considered suitable for Cu-B-CrC composites. Due to its softer structure, Cu produces a smaller coefficient of friction compared to reinforced samples. Thus, with the increasing hardness of the reinforced materials, the coefficient of friction demonstrates a decreasing trend with the same reason on weight loss and wear rate.

**Figure 16:**
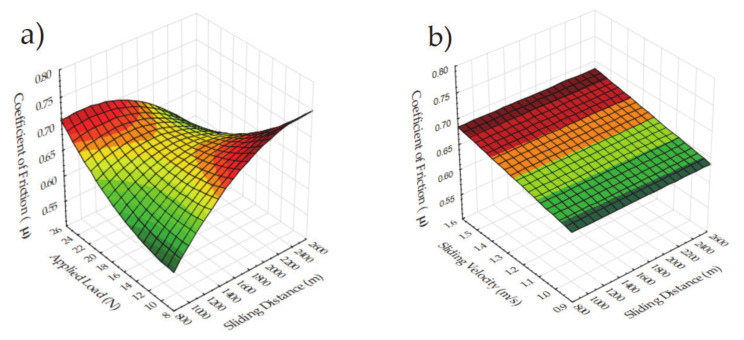

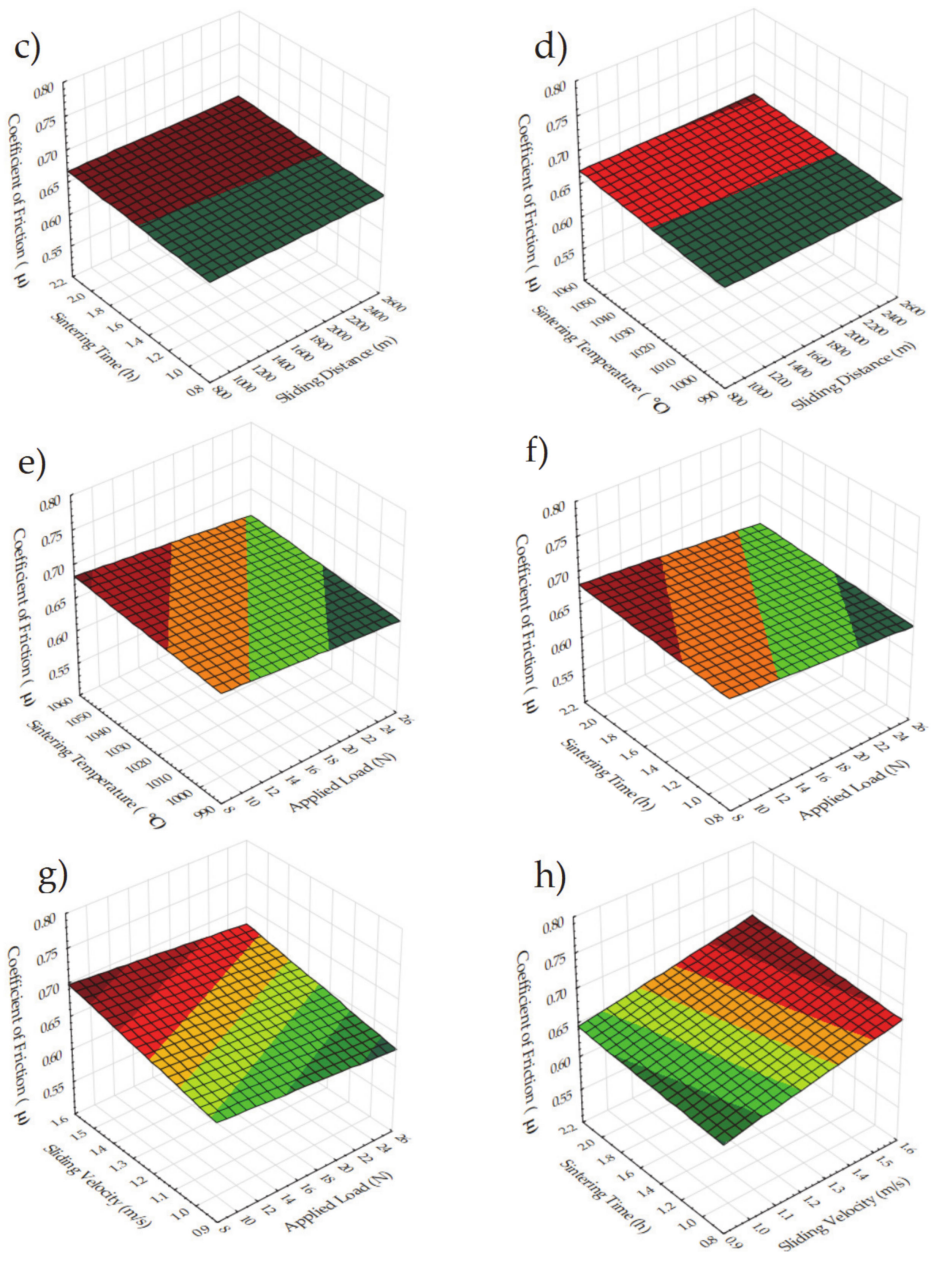

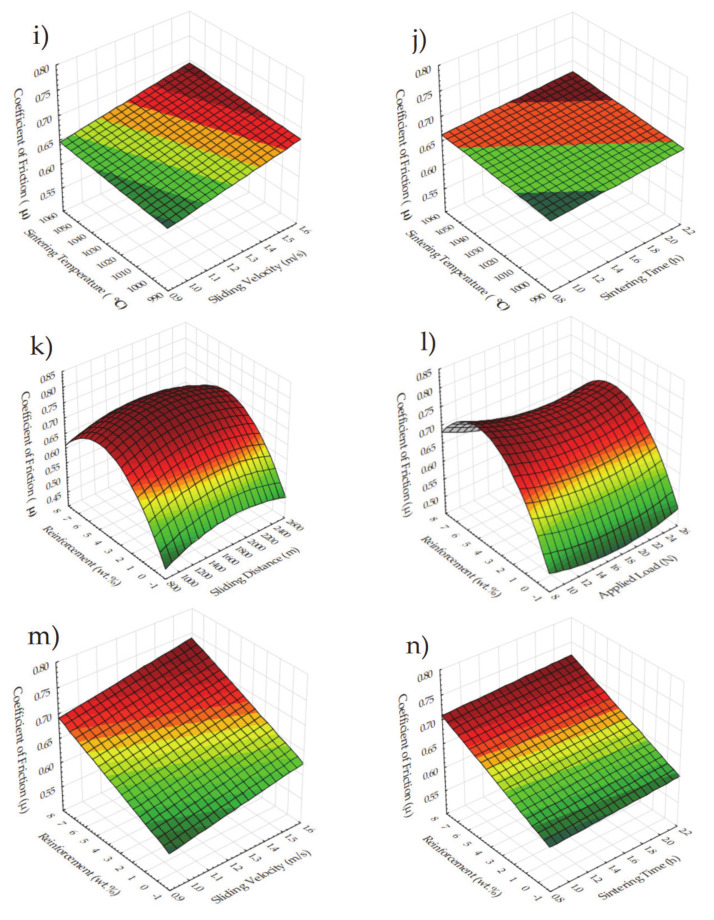

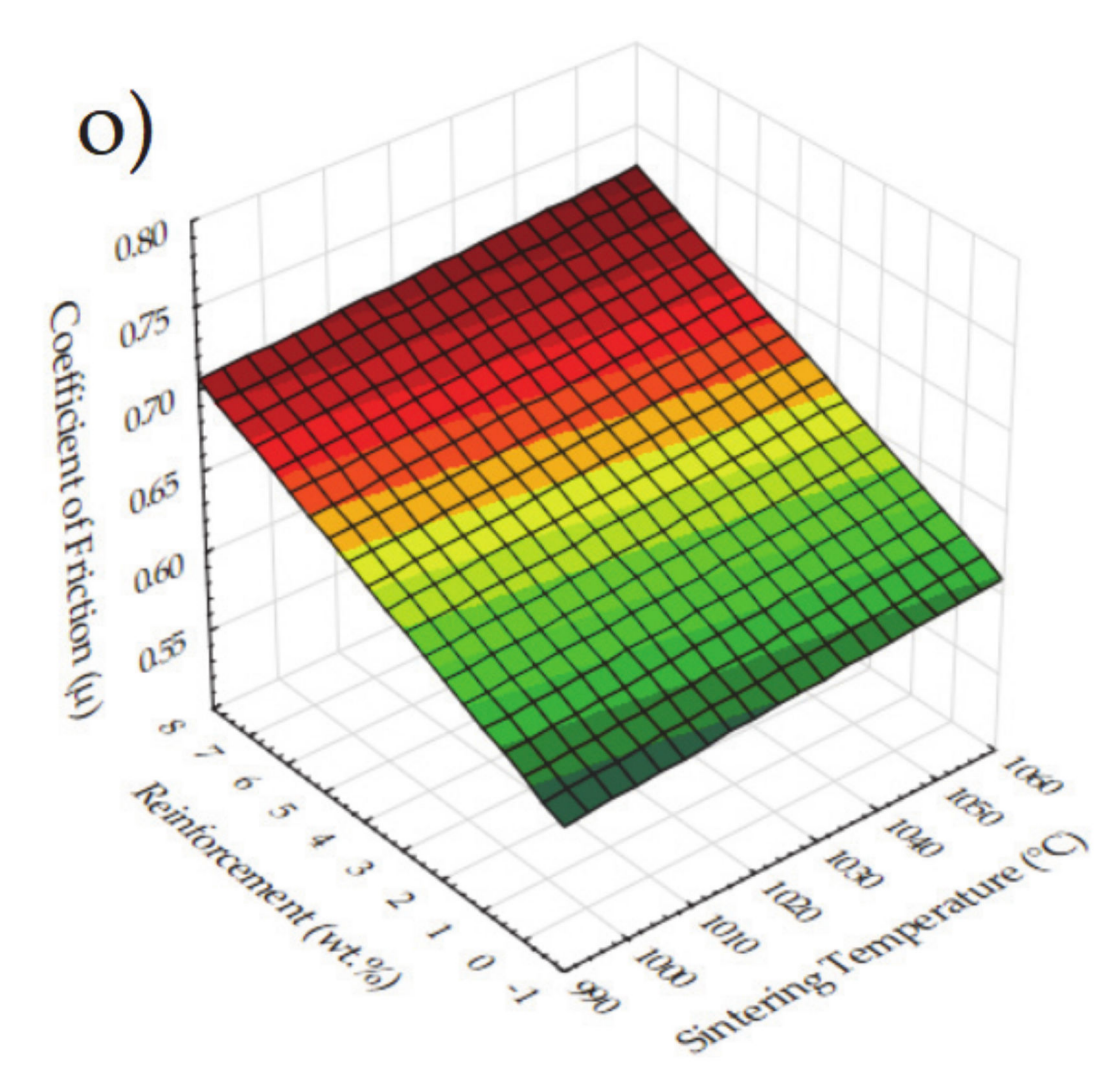
3D surface plots for the coefficient of friction for the effect of (**a**) Sliding distance and applied load (**b**) Sliding velocity and sliding distance (**c**) Sintering time and sliding distance (**d**) Sintering temperature and sliding distance (**e**) Sintering temperature and applied load (**f**) Sintering time and applied load (**g**) Sliding velocity and applied load (**h**) Sintering time and sliding velocity (**i**) Sintering temperature and sliding velocity (**j**) Sintering temperature and sintering time (**k**) Reinforcement and sliding distance (**l**) Reinforcement and applied load (**m**) Reinforcement and sliding velocity (**n**) Reinforcement and sintering time (**o**) Reinforcement and sintering temperature.

As it can be seen in Figure 16, colored surfaces reflect the effectiveness of the design parameters on the coefficient of friction. Especially for the sliding distance versus sintering properties, no important change can be seen in Figure 16c,d. On the other hand, slight changes are observed for many parameters, for example, in Figure 16e,f,j. Owing to the dominance of reinforcement on the coefficient of friction, these parameters stay in the background. It is seen with the increase in the sliding velocity, the coefficient of friction demonstrates increasing behavior (Figure 16b,g,h,I,m). Sintering parameters have no influence on the coefficient of friction at all (Figure 16c–f,h–j,n,o). Despite the fact that the applying load has no major influence on the combinations of other parameters, sliding distance makes it important according to Figure 16a. Therefore, this combination needs to be considered in future studies. Lastly, the reinforcement ratio makes the maximum variation in coefficient of friction as it can be seen in Figure 16k,l, and 13m–o. In the quadratic models, the curve for the high reinforcement samples indicates a better coefficient of friction conditions. Lastly, it can be noted that reinforcement is the dominant parameter, followed by tribological parameters. Additionally, sintering parameters have much less effect on the coefficient of friction.

### 3.6. ANOVA Results for Wear Rate, Weight Loss and Coefficient of Friction

ANOVA is a widely preferred statistical analysis method that gives the efficiency of the design parameters on the quality characteristics [75,76]. In this work, wear rate, weight loss and coefficient of friction were taken into account as quality parameters while production and tribology parameters mentioned before were presented as the design parameters or sources. The main objective here is to calculate the degree of effectiveness of each source with several statistical values. The sum of squares (SS) is calculated by the difference between the mean value and the result of each experiment. Despite the fact that there is a sum of impact for each source, the remaining values from them describe the error value which can be accepted as the undetermined factor. The mean square (MS) is calculated by dividing the SS value by the degree of freedom (DOF). Then, the F value is found by dividing the SS value of each parameter by the error value. An important parameter, *p*-value, shows if a parameter is statistically important or not in the confidence interval (95%). This implies that if the value is smaller than 5%, then it is important at the range of the confidence interval. The last parameter is the percent contribution (PC%) that is calculated by dividing each SS value by the total SS. 

Table 3 outlines the ANOVA results for signal-to-noise ratios of wear rate, weight loss and coefficient of friction, respectively. When looking at the PC values, reinforcement is the dominant parameter on wear rate (51.6%) and the coefficient of friction (79.9%). In addition, according to *p*-values, reinforcement affects the coefficient of friction (0.048 < 0.05). It is noteworthy to mention that for better *p* values, a higher number of samples need to be considered for weight loss and wear rate. Weight loss seems to be affected by the sliding distance (36.7%) first, followed by the reinforcement ratio (27.25%) and applied load (24.5%), respectively. The applied load also seems efficient on the wear rate, with a high PC value (30.07%). F values confirm the dominance of the mentioned parameters. Accordingly, except for the sorted parameters, other ones can be ignored at least for the statistical evaluation. However, the total effect of the remaining parameters reveals a considerable impact that needs to be considered with further analysis. It can be said that from the production process to the tribological tests, effective parameters have been included, which is understood from the low values of errors (4.44%, 3.15% and 8.3%). In a nutshell, the tribological performance is affected mostly by the sliding distance, applied load and reinforcement ratio.

## 4. Conclusions

This paper investigates the effect of process parameters used in the production of Cu-B-CrC composites, namely reinforcement ratio, sintering time and sintering temperature and tribological parameters, i.e., sliding velocity and applied load, to evaluate the tribological performance of the composite in terms of its coefficient of friction, wear rate and weight loss. The Taguchi design of experiment and ANOVA analysis were performed to further analyze the effect of process parameters on the analyzed outputs. The current study provides significant information about the real-life conditions of Cu-B-CrC composites in terms of its tribological performance. The following conclusions can be made from the analysis and evaluation of the Cu-B-CrC composites:

1. As a result of the density and hardness tests of the composites sintered at different times and temperatures, the highest relative density value was determined as 93.4%. The increase in sintering time and temperature caused an increase in its density. The highest hardness value was determined as 67.48 HB in the 7.5 wt.% reinforced sample (at 1050 °C for 1 h). It was observed that the increase in reinforcement ratios affected the hardness positively. In addition, the lowest hardness value was observed in the pure Cu sample with approximately 45.5 HB.
2. As a result of the SEM analysis, it was observed that the pores decreased with the reinforcement particles and the density increased accordingly. In addition, it was observed in SEM analysis that the added B-CrC reinforcement elements were homogeneously distributed.
3. According to signal-to-noise ratios, at conditions of 2500 m, 10 N, 1.5 m/s, 2 h, 1050 °C and 0 wt.%, the minimum wear rate can be obtained. Similarly, at 1000 m, 10 N, 1.5 m/s, 2 h, 1050 °C and 2.5 wt.% and 1000 m, 15 N, 1 m/s, 1 h, 1000 °C and 0 wt.% conditions are recommended for minimal weight loss and a reduced coefficient of friction.
4. From ANOVA results, it is understood that among the input parameters, the reinforcement ratio has considerable influence on the wear rate (51.6%), weight loss (27.25%) and coefficient of friction (79.9%). The applied load is one of the important parameters that affect the wear rate (30.07%) and weight loss (24.5%), respectively. It should be noted that sliding distance is the most effective parameter on weight loss (36.7%). In addition, other parameters except the mentioned ones have a small effect that can be ignored.
5. R-square values of the statistical analysis demonstrate that the effective parameters have been successfully detected for each result (wear rate—95.56%, weight loss—96.85% and coefficient of friction—91.7%). It is important since, from the design stage of the composite material, influential factors can be determined. This provides a tool which assists in the production of durable materials for specific applications.

## Figures and Tables

**Table 1:** The properties of powder materials.

Samples	Cu Ratio (wt.%)	B Ratio (wt.%)	Cr_3_C_2_ Ratio (wt.%)	Number of Samples	New Representation
Pure Cu	100	–	–	3	Cu
Cu-B-Cr_3_C_2_ (2.5 wt.%)	97.5	1	1.5	3	Cu-B-1.5-Cr_3_C_2_
Cu-B-Cr_3_C_2_ (5 wt.%)	95	2	3	3	Cu-2B-3Cr_3_C_2_
Cu-B-Cr_3_C_2_ (7.5 wt.%)	92.5	3	4.5	3	Cu-3B-4.5 Cr_3_C_2_

**Table 2:** Design parameters and related experimental results after the tribological tests.

Exp. No	Reinforcement Ratio (wt.%)	Sliding Distance (m)	Applied Load (N)	Sliding Velocity (m/s)	Sintering Time (h)	Sintering Temperature (°C)	Weight Loss (g)	Wear Rate (×10^−4^ mm^3^/Nm)	Coefficient of Friction (µ)
1	0	1000	10	1	1	1000	0.007600	9.49701	0.512543
2	0	1500	15	1	1	1050	0.016000	12.76947	0.553592
3	0	2000	20	1.5	2	1000	0.018100	10.90451	0.585360
4	0	2500	25	1.5	2	1050	0.023800	11.37495	0.557652
5	2.5	1000	15	1.5	2	1000	0.007200	9.47045	0.754312
6	2.5	1500	10	1.5	2	1050	0.006300	5.56853	0.749834
7	2.5	2000	25	1	1	1000	0.028700	18.96008	0.691095
8	2.5	2500	20	1	1	1050	0.029900	15.66025	0.708838
9	5	1000	20	1	2	1050	0.017900	26.81273	0.641499
10	5	1500	25	1	2	1000	0.038500	36.7995	0.707800
11	5	2000	10	1.5	1	1050	0.031300	23.43129	0.787704
12	5	2500	15	1.5	1	1000	0.033500	18.63495	0.64229
13	7.5	1000	25	1.5	1	1050	0.014400	21.53336	0.68217
14	7.5	1500	20	1.5	1	1000	0.028300	28.7164	0.667579
15	7.5	2000	15	1	2	1050	0.020200	15.21726	0.658962
16	7.5	2500	10	1	2	1000	0.016500	9.82809	0.671620

**Table 3:** Analysis of variance for signal-to-noise ratios of wear rate, weight loss and coefficient of friction.

Source	DOF	SS	MS	F Value	*p*-Value	PC (%)
*Wear Rate*						
Sliding Distance	3	8.659	2.886	0.34	0.801	3.04
Applied Load	3	85.649	28.550	3.34	0.137	30.07
Sliding Velocity	1	5.721	5.721	0.67	0.459	1.85
Sintering Time	1	25.156	25.156	2.94	0.162	8.83
Sintering Temperature	1	0.489	0.489	0.06	0.823	0.17
Reinforcement	3	146.476	48.8252	8.22	0.110	51.6
Error	3	12.687	4.2291	-	-	4.44
Total	15	284.837	-	-	-	100
*Weight Loss*						
Sliding Distance	3	132.303	44.1011	4.70	0.085	36.7
Applied Load	3	88.347	29.4491	3.14	0.149	24.5
Sliding Velocity	1	5.243	5.2434	0.56	0.496	1.4
Sintering Time	1	24.858	24.8576	2.65	0.179	6.9
Sintering Temperature	1	0.686	0.6861	0.07	0.800	0.1
Reinforcement	3	97.947	32.6491	8.59	0.055	27.25
Error	3	11.397	3.7989	-	-	3.15
Total	15	360.782	-	-	-	100
*Coefficient of Friction*						
Sliding Distance	3	0.6421	0.21403	0.07	0.971	3.9
Applied Load	3	0.2379	0.07929	0.03	0.993	1.4
Sliding Velocity	1	0.8345	0.83445	0.29	0.621	5
Sintering Time	1	0.1021	0.10208	0.03	0.861	0.8
Sintering Temperature	1	0.1151	0.11515	0.04	0.852	0.7
Reinforcement	3	13.1271	4.37570	9.58	0.048	79.9
Error	4	1.3703	0.45677	-	-	8.3
Total	15	16.4290	-	-	-	100

## Data Availability

Not applicable.
